# 3D-Printed Concentration-Controlled Microfluidic Chip with Diffusion Mixing Pattern for the Synthesis of Alginate Drug Delivery Microgels

**DOI:** 10.3390/nano9101451

**Published:** 2019-10-12

**Authors:** Shixuan Cai, Hongyan Shi, Guoqian Li, Qilu Xue, Lei Zhao, Fu Wang, Bo Hu

**Affiliations:** 1 School of Life Science and Technology, Xidian University, Xi’an 710126, Shaanxi, China; sxcai@stu.xidian.edu.cn (S.C.); hyshi@xidian.edu.cn (H.S.); guoqianli@stu.xidian.edu.cn (G.L.); xueql@stu.xidian.edu.cn (Q.X.); 2 Kunpad Communication Pty. Ltd., Kunshan 215300, Jiangsu, China

**Keywords:** 3D-printing, microfluidic chips, concentration controlled, diffusion mixing pattern, calcium alginate microgels

## Abstract

Alginate as a good drug delivery vehicle has excellent biocompatibility and biodegradability. In the ionic gelation process between alginate and Ca^2+^, the violent reaction is the absence of a well-controlled strategy in the synthesizing calcium alginate (CaA) microgels. In this study, a concentration-controlled microfluidic chip with central buffer flow was designed and 3D-printed to well-control the synthesis process of CaA microgels by the diffusion mixing pattern. The diffusion mixing pattern in the microfluidic chip can slow down the ionic gelation process in the central stream. The particle size can be influenced by channel length and flow rate ratio, which can be regulated to 448 nm in length and 235 nm in diameter. The delivery ratio of Doxorubicin (Dox) in CaA microgels are up to 90% based on the central stream strategy. CaA@Dox microgels with pH-dependent release property significantly enhances the cell killing rate against human breast cancer cells (MCF-7). The diffusion mixing pattern gives rise to well-controlled synthesis of CaA microgels, serving as a continuous and controllable production process for advanced drug delivery systems.

## 1. Introduction

Alginate microgels have a wide variety of pharmaceutical and biomedical applications, such as delivery vehicles of natural medicine [1,2], liquid metal droplets [3], protein [4,5], hydrophilic and hydrophobic drugs [6], MR imaging [7], cell encapsulation [8,9] and biocatalyst [10], due to its advantageous features of biocompatibility, low toxicity [11], low cost, magnetic property, controlled release [12] and stimuli-response [13,14]. Alginate hydrogels have three synthesis methods, including ionic cross-linking gelation, covalent cross-linking gelation and thermal gelation, in which ionic cross-linking gelation is the most frequently-used method [15]. However, high solubility of calcium ions in aqueous solution will lead to a poorly controlled preparation of alginate hydrogels. When the reactivity of ionic cross-linkers (Ca^2+^) is reduced, the cross-linking process becomes slower, and the properties of hydrogels are much better [16]. Thus, gelation rates have a critical effect on the gelation process. The buffer containing phosphate (e.g., sodium hexametaphosphate), insoluble divalent salts (e.g., CaCO_3_, CaSO_4_) and reaction temperature, have been used to control the gelation process [17]. These methods will introduce new impurities, leading to additional purification processes. Further, the environmental pH and temperature might change, leading to lower system stability. Therefore, there is growing need for the development of a controlled synthesis method of alginate microgels.

Microfluidic platforms can precisely control over fluid flow in the microchannels [18]. Microfluidic chips have synthesized nano/micro-hydrogels, which have a wide range of biological and medical applications, such as enzyme-catalyzed reactions [19], cell manipulation [20], tumor screening [21] and microparticle synthesis [22,23,24,25]. Hydrodynamic flow focusing in straight or modular microfluidic platforms was mostly utilized for producing microparticles. Some studies have demonstrated that flow focusing in microfluidic chip can produce nanoparticles in a narrow distribution [26,27]. Amanda C. S. N. Pessoa et al. have used a central aqueous stream configuration to successfully synthesize chitosan nanoparticles based on an ionic crosslinking manner [28,29]. Adam Bohr et al. have demonstrated that polyelectrolyte complexation can prepare alginate microgels with small sizes by microfluidic technology [30]. 3D-printed micromixers have employed the convective mixing process for the synthesis of alginate microgels with particle sizes of around 200 nm [31,32]. However, the convective mixing process is too intense and rapid. It may lead to a bulky gelation of alginate hydrogels and microchannel blocking. Currently, the synthesis of alginate microgels in a diffusion mixing pattern is still out of reach.

Here, a 3D-printed, concentration-controlled, microfluidic chip for manufacturing alginate microgels with diffusion mixing pattern was designed to solve the problems of the rapid ionic gelation rate between alginate and free cations. The diffusion mixing pattern can slow down the gelation rate in the central stream. Microfluidic chip length and flow rate ratio have been studied. Calcium alginate (CaA) microgels can efficiently load doxorubicin (Dox), which has been used for breast cancer chemotherapy in vitro.

## 2. Materials and Methods

### 2.1. Materials

Sodium alginate was purchased from Sigma-Aldrich (Saint Louis, MI, USA). Calcium chloride anhydrous was purchased from Aladdin. Doxorubicin (Dox) was purchased from Sigma. Deionized water was used for all experiments.

For cell experiments, Trypsin was purchased from Corning cellgro. Fetal bovine serum, penicillin/streptomycin were purchased from Aladdin. High-glucose Dulbecco’s modified Eagle’s medium (DMEM) was purchased from Nalgene (Milwaukee, WI, USA). CCK-8 kit was purchased from DoJinDo (Shanghai, China). Fluorescein diacetate (FDA) was purchased from MP Biomedicals (Santa Ana, CA, USA). Propidium iodide (PI) was purchased from Sangon (Shanghai, China).

### 2.2. Microfluidic Devices Design and 3D-Printed Fabrication

The microfluidic chip for preparing CaA@Dox or CaA microgels has three inlets and three outlets (1 mm width × 0.2 mm depth × 5 mm length), one straight mixing channel (1 mm width × 0.2 mm depth). Four microfluidic chips with length of 30 mm, 40 mm, 50 mm, and 100 mm were used in this work (Figure S1).

All microfluidic chips were designed and drawn by SolidWorks (SolidWorks 2016) in STL format and printed via a Projet 3500 HD MAX printer of 3D systems (Rock Hill, CA, USA) with Visjet^®^ M3 Crystal build materials and Visjet^®^ R S300 sacrificial materials.

### 2.3. Inner and Outer Sacrificial Materials Removal

The outer sacrificial materials of microfluidic chips were removed by heating in 70 °C for 10 min. The inner sacrificial materials was removed successively by vegetable oil of 70 °C for 30–60 min, and deionized water for 1 h. A constant flow pump (Shanghai QiTi analytic instrument Inc., Shanghai, China) was used to pump removers (vegetable oil) into the inner chip channels with the rate of 0.1–0.3 mL/min [33,34].

### 2.4. Numerical Simulations

Numerical simulation was used to investigate the diffusion and mixing process in the complex microchannels. The fluid dynamic and concentration fields were calculated by a commercial analysis software, COMSOL Multiphysics 5.3a (COMSOL Inc., Stockholm, Sweden). The Laminar Flow module (spf) and Transport of Diluted Species module (tds) were employed, and the study was set as “Stationary”. The mesh quality was set as “Finer” controlled by the physical field.

During the simulation, the fluid flow in the channels was solved with the incompressible Navier-Stokes equations as shown.

(1) $$\rho \frac{\partial u}{\partial t}-\nabla \cdot \left[-pI+\eta \left(\nabla u+{\left(\nabla u\right)}^{T}\right)\right]+\rho u\cdot \nabla u=F$$

(2) −∇⋅u=0,

where *ρ* is fluid density, *u = (u, v, w)* is the flow-velocity field, *p* is fluidic pressure, *I* is the unit diagonal matrix, *η* is the dynamic viscosity, and *F = (f_x_, f_y_, f_z_)* is a volume force affecting the fluid. The fluid is water with *ρ* = 1,000 kg/m^3^, *η* = 0.001 Pa·s, and *F* = 0 because there are no volume forces.

In addition, the concentration of the dissolved substances in the fluid was described by the following convection-diffusion equation.

(3) $$\frac{\partial c}{\partial t}+\nabla \cdot \left(-D\nabla c\right)=R-u\cdot \nabla c$$

where *c* is the concentration, *D* is the diffusion coefficient, and *R* is the reaction rate. In this model, *D* = 1×10^−9^ m^2^/s, and *R* = 0 because the concentration is not affected by any reactions.

### 2.5. Definitions to Evaluate the Mixing Process

Firstly, the Flow Rate Ratio (*R_f_*) was defined as the flow rate of two side streams (*F_s_*) over the flow rate of middle stream (*F_c_*), as shown in the following equation.

(4) $$R_{f}=\frac{\sum F_{s}}{F_{c}}$$

Different *R_f_* values during synthesis of CaA microgels were aimed to investigate the effects of the flow fields on concentration variation rate and particle size.

Secondly, for each microfluidic device, concentration profile was obtained at the microchannel cross-section by COMSOL software. Mixing efficiency (ME) was calculated by minimum and maximum variance (*σ*) of the concentration in microfluidic cross-section.

(5) $$ME=1-\sqrt{\frac{\sigma_{min}}{\sigma_{max}}}$$

where σ_*min*_, σ_*max*_ are the concentration variance of minimum and maximum. *ME* = 0 and *ME* = 1 indicate complete unmixing and mixing.

Thirdly, the Mixing Time (*t_mix_*) indicates the time that solution distributes uniformly across the cross-section of the channel. It was calculated by the following equation.

(6) $$t_{mix}=\;\frac{W^{2}}{9D\;\;{\left(1+R_{f}\right)}^{2}}$$

where *W* and *D* was channel width and the diffusion coefficient. *R_f_* was flow rate ratio.

Finally, the Resident Time (*t_r_)* was defined as the time that solution goes through channel by the following equation.

(7) $$t_{r}=\frac{L}{F/S}$$

where *L*, *F*, *S* indicate length, total flow rate of channel and cross section area.

### 2.6. Fluorescence Assay

Fluorescence assays for the diffusion and mixing efficiency verification were performed in a microfluidic chip of 100 mm length at flow rate ratio (*R_f_*) of 0.5, 1, 2 and 4. For the side inlet channels, two syringes were loaded with 10 μM fluorescein solution, and one syringe with deionized water was used for the central inlet channel. Syringe pumps (ERA-Syringe Pump, USA) were used to adjust the flow rates of the central water streams (*F_c_*) and side dye streams (*F_s_*). The total flow rate (*F*) was a constant of 600 μL/min. The fluorescence images were taken with an inverted microscope (Inverted Research Microscope Eclipse Ti-U, Nikon, Tokyo, Japan). Data was analyzed by MATLAB to extract the grayscale value of fluorescence pictures.

### 2.7. Synthesis of CaA and CaA@Dox Microgels

CaA and CaA@Dox microgels were produced on these parameters: total flow rate (*F*) of 600 μL/min, sodium alginate and CaCl_2_ concentration of 0.1% (*w*/*v*, g/mL), equal side stream flow rate (*Fs*).

Before the microfluidic fabrication of nanoparticles, sodium alginate, CaCl_2_ and Dox were dissolved in deionized water in the desired concentrations. While sodium alginate was kept overnight under magnetic stirring, and CaCl_2_ was freshly prepared.

**Figure 1:**
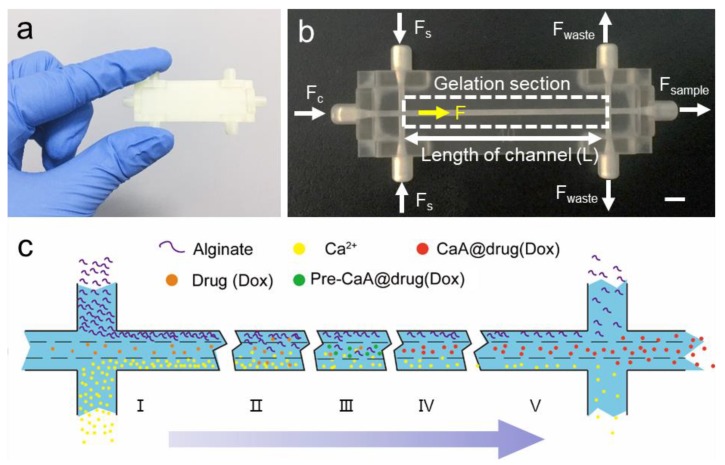
3D-printed concentration-controlled microfluidic chip for the synthesis of CaA microgels and the diffusion mixing pattern. (**a**) Photograph of the 3D-printed concentration-controlled microfluidic chip. (**b**) Photograph of the 3D-printed concentration-controlled microfluidic chip of 30 mm. The *F_c_* represents the middle stream flow rate. *F_s_* represents the side stream flow rate. F represents the total flow rate in channels. The scale bar is 3 mm. (**c**) Schematic representation of the diffusion mixing pattern where the large differential concentration drives the diffusion of alginate molecules and Ca^2+^ ions into the central stream, and later gradually generating microparticles when traveling along the microchannels. The formation of products is divided into the following steps: entrance, diffusion, mixing, gelation, collection.

To prepare CaA or CaA@Dox microgels, syringe pumps were employed to introduce the sodium alginate and CaCl_2_ through two side inlets, while water went through the middle inlet simultaneously (Figure 1). Productions were collected from middle outlet finally.

### 2.8. Nanoparticle Morphology

Size distribution, particle size and the zeta potential of CaA and CaA@Dox microgels were measured by Zetasizer Nano ZS (Marvern, USA). TEM was performed on a JEOLFETEM-2100 transmission electron microscope (JEOL, Japan) under 200 kV accelerating voltage. UV-vis absorption spectroscopy was obtained on TU-1900 spectrophotometer (Pgeneral, China). The concentration of the CaA microgels was measured by weighing the dried suspensions after freeze drying for 24 h.

### 2.9. In vitro Release of Dox

In vitro release of Dox from the CaA microgels was performed in phosphate buffer saline (PBS) of pH 7.4 and pH 6.5 at 37 °C. Briefly, 700 μL CaA@Dox microgels (20 μg/mL) were placed in a dialysis bag (MW14000) which was put into 120 mL PBS and shaken (100 rpm) at 37 °C. Fresh medium was replaced at predetermined times and we collected the medium for a UV measurement (absorbance at 480 nm). The release rate at each time was calculated as the sum of free Dox in the solution over total Dox before dialysis.

### 2.10. Cell Culture

MCF-7 and MC7-10A were grown in an incubator at 37 °C with 95% air and 5% CO_2_ in DMEM supplemented with 10% fetal bovine serum and 1% penicillin-streptomycin solution.

### 2.11. In vitro Cytotoxicity

The in vitro cytotoxicity of empty CaA microgels and Dox loaded CaA microgels were evaluated by standard CCK-8 kit assays. MCF-7 and MCF-10A were used. Cells were seeded in a 96-well plate at a density of 5 × 10^4^ cells (100 μL) per well and incubated for 24 h.

Each well was washed three times with PBS mildly. The serial concentrations of empty CaA microgels, Dox-loaded CaA microgels, free Dox and deionized water at same volume were added, respectively. Two rows of 96-well plate were used as a control with 100 μL culture medium only, and cells in medium, respectively. After incubation for 24 h, 48 h and 72 h, 10 μL CCK-8 solution was added to each well and there was incubation for another 1 h. The absorbance was monitored with a GloMax^®^ Discover microplate reader (Promega, USA) at a wavelength of 450 nm. Cell relative viability was determined by the following equation.

(8) $$\mathrm{Relative}\;\mathrm{Cell}\;\mathrm{Viability}\;\left(\%\right)=\frac{Abs_{\left(test\;well\right)}-Abs{\;}_{\left(\;medium\;only\right)}}{Abs_{\left(cell\right)}-Abs{\;}_{\left(\;medium\;only\right)}}\;\times 100\%$$

### 2.12. Live/dead Assay

After incubating cells with CaA microgels, Dox loaded CaA microgels, free Dox and deionized water for 24 h, 1 μL PI (5 μg/mL) and FDA (5 μg/mL) in medium for each well were added. Then incubated for another five minutes under the conditions of protection from light. The fluorescence images were taken with an inverted microscope.

## 3. Results and Discussion

### 3.1. Design of 3D-Printed Concentration-Controlled Microfluidic Chip

The concentration-controlled microfluidic chip was investigated to synthesize CaA microgels in a continuous way. The microfluidic chip consists of three inlets, three outlets, and one straight channel (Figure 1a,b). It adopts a central flow structure with three tributaries to realize diffusion control and gelation of ions. CaA microgels are prepared through controlled diffusion and mixing between one water stream in the middle inlet channel and two reagent streams in the side inlet channels. When the total flow rate keeps constant, the flow rate ratio can be well-controlled, which was defined as the flow rate of two side streams over the flow rate of middle stream. The process of ionic gelation was mainly carried out in the central stream. CaA microgels were collected from central outlet. Model graph designed by Solidworks and photographs of chips investigated in this study are shown in Figure S2.

The schematic image of the concentration-controlled microfluidic chip is shown in Figure 1c. A central stream structure has been used to slow down gelation process. Microfluidic chip as an integrated region consists of three continuous sections, including entrance section, gelation section and export section. The entrance section is to inject reagent and control reaction conditions. The gelation section is to prepare CaA microgels (or CaA@drug microgels). The export section has one middle outlet to collect product and two side outlets to take out unreacted reagents.

The gelation section consists of two consecutive steps. Firstly, the large differential concentration drives the diffusion of alginate molecules and Ca^2+^ ions into the central stream. Secondly, the gelation process was carried out with the crosslinking reaction between alginate molecules and Ca^2+^ ions to form CaA microgels. There are no strict limits between these two consecutive steps. Initially, the diffusion process is dominant. Later, the mixing process and gelation process can act simultaneously. The central stream structure generates a transition region for crosslinking and slowing down the gelation rate. The concentration-controlled diffusion of alginate molecules and Ca^2+^ ions have a significant effect on the gelation rate, reaction rate and properties of microgels.

### 3.2. Concentration-Controlled Process

**Figure 2:**
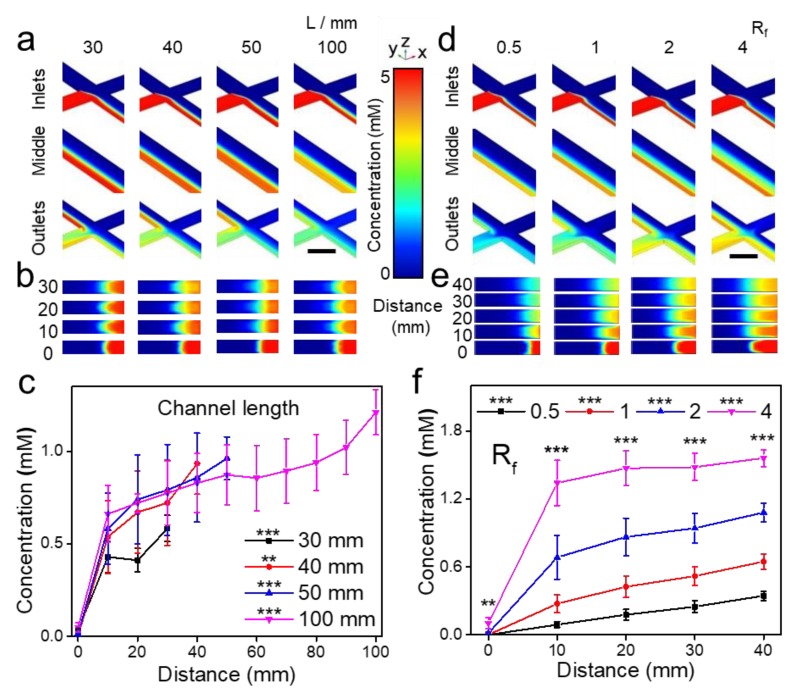
COMSOL simulation to show the impact of channel length and flow rate ratio (*R_f_*) on the concentration-controlled process. To explore the impact of channel length: (**a**) Concentration distribution of inlets, middle site and outlets of microfluidic chips with lengths of 30, 40, 50, 100 mm. (**b**) Concentration distribution of cross sections at the distances of 0, 10, 20, 30 mm away from inlets. (**c**) Calculate the concentration distribution of cross sections at the distances of 0, 10, 20, 30 mm away from inlets in microfluidic chips of 30, 40, 50, 100 mm. To explore the impact of *R_f_*: (**d**) Concentration distribution of channel inlets, middle site and outlets under the flow rate ratio of 0.5, 1, 2, 4. (**e**) Concentration distribution of the cross section at the distances of 0, 10, 20, 30 mm away from inlets. (**f**) Calculate the concentration distribution of cross section at the distances of 0, 10, 20, 30 mm away from inlets under flow rate ratio of 0.5, 1, 2, 4. Data are shown as mean± SEM, n > 50, * *p* < 0.05, ** *p* < 0.01, *** *p* < 0.001.

To investigate how to manipulate the concentration-controlled process, a multi-physics simulation software COMSOL was used to calculate concentration of sodium alginate and Ca^2+^ ions in channels under steady state (details in Materials and methods). The average concentration at the central line of the cross-section perpendicular to stream direction was calculated to represent the diffusion process of ions. The error bar represents standard deviation of concentration throughout the central lines. Four different channel lengths were used to investigate the concentration distribution and diffusion (Figure 2a–c). The obvious diffusion and incomplete mixing could be seen in the microfluidic chip with different channel length (Figure S3a–d). Cross sections at sites of 0, 10, 20, 30 mm away from the inlets had shown that the diffusion from side to central were significant (Figure 2b). While chips lengthened from 30 mm to 100 mm, the diffusion from side to central increased slowly (Figure 2c).

Chip width and depth were investigated to explore their impacts on the diffusion process (Figure S4). For the chip width from 0.6 mm to 1.0 mm, the average concentration of alginate at the central line of the cross-section displayed an increased relationship regarding the length in the range of 0–100 mm. The chip width of 1.0 mm had the highest average concentration. For the chip depth from 0.2 mm to 0.3 mm, the average concentration had the similar increasing trend regarding the length in the range of 0–100 mm. The chip depth of 0.3 mm had the highest average concentration. The gentlest mixing process was observed when the depth and width are 0.2 mm and 1 mm, respectively.

The effects of the flow rate ratio (*R_f_*) on the concentration distribution and diffusion were studied (Figure 2d–f). When *R_f_* increased from 0.5 to 4, the diffusion of sodium alginate in side stream was significant (Figure S3a–d). *R_f_* of 4 had the highest concentration and increased slowly after the length of 10 mm. Further, *R_f_* was defined as the flow rate of two side streams over the middle stream. Increasing *R_f_* may lead to more of those reactants adding into the channel and narrower middle reaction area, which makes the diffusion process not obvious. Therefore, lower *R_f_* are expected to be used for the dominant diffusion process.

The total flow rate can further affect the concentration distribution. As the total flow rate increased from 150 to 600 μL/min, the diversity of concentration at different distances were similar (Figure S5). Therefore, total flow rate has no obvious impact on concentration distribution.

### 3.3. Fluorescence Assay

**Figure 3:**
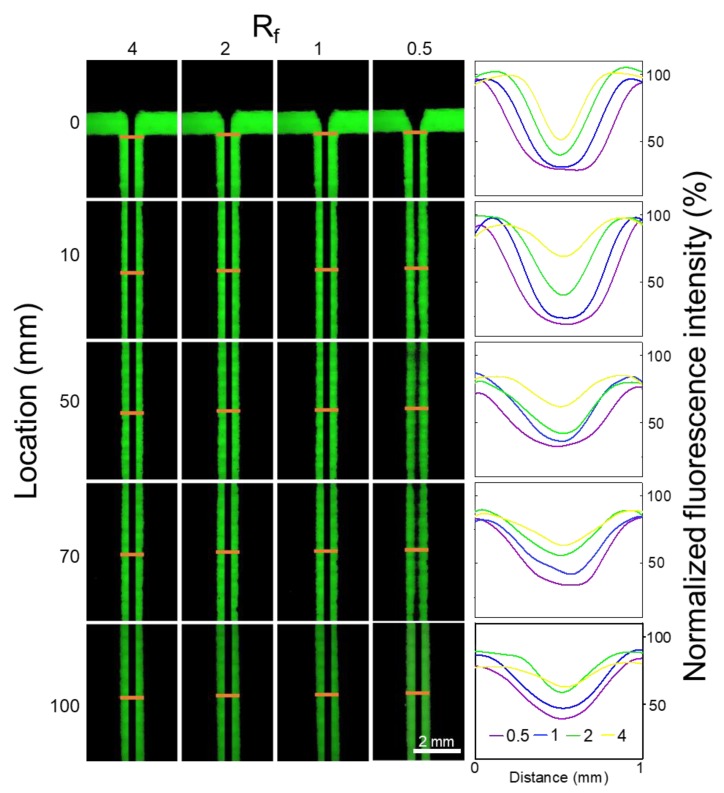
Fluorescence assays performed to investigate the diffusion process of two side streams composed of 10 μM fluorescein through a 3D-printed concentration-controlled microfluidic device. Chips with four flow rate ratios (*R_f_*) of 0.5, 1, 2, 4 were explored. Fluorescent microscopy images were taken at distances of 0, 10, 50, 70, 100 mm away from the inlets of channels. The yellow lines indicate fluorescence intensity profiles at the corresponding locations. X and Y represent the relative channel width and fluorescence intensity, respectively. Scale bar was 2 mm.

To investigate the diffusion and mixing process of the ions in the channels, fluorescence assays using fluorescein were performed to mimic the alginate and CaCl_2_ streams (Figure 3). The fluorescence microscopy images were taken along the microchannel at the distances of 0, 10, 50, 70 and 100 mm away from inlets, respectively.

A customized image processing algorithm based on commercial MATLAB software was used to calculate the relative fluorescence intensity along channels at different distances. In the 3D-printed concentration-controlled microfluidic chip with diffusion mixing pattern, the channel presented stable and continuous laminar flow structure. The lower fluorescence intensity at the middle of the channel represents that fluorescein diffused from the side of the channel to the central water stream and the steam’s complete mixing was not achieved. The fluorescence intensity increased along the channel length (Figure S6). When *R_f_* increased from 1 to 4, the fluorescence intensity increased accordingly. *R_f_* of 4 had the highest fluorescence intensity. It is interestingly noted that the central stream become narrower as the *R_f_* increased, and the relative fluorescence intensity at middle site was bigger (Figure S6).

To reveal the mixing process, the mixing efficiency was investigated by COMSOL (Tables S1 and S2). The theoretical mixing time for channels with *R_f_* of 0.5, 1, 2, 4 were 49.38, 27.78, 12.35 and 4.44 s, respectively (Table S2). Resident time that streams flow through channels was 0.6, 0.8, 1.0, and 2.0 s, respectively. Mixing time was much longer than resident time. Thus, the mixings of reagents along the channel length were not complete. The incomplete mixing may be attributed to a wide channel. Meanwhile, the relationship between channel length and mixing efficiency represented a good linear relationship (Figure S7).

### 3.4. Synthesis of CaA Microgels

**Figure 4:**
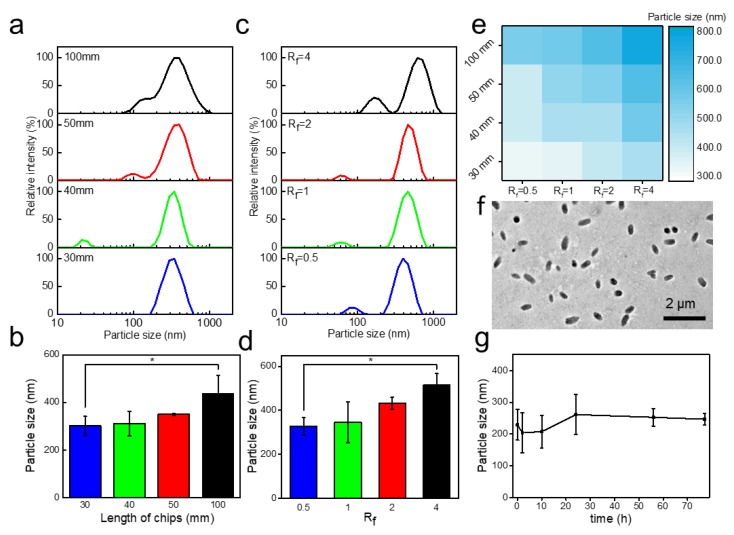
3D-printed concentration-controlled microfluidic chip-based fabrication of CaA microgels. (**a**) Hydrodynamic sizes intensity distribution of CaA microgels with different channel lengths measured by DLS. (**b**) Hydrodynamic particle sizes of CaA microgels with channel lengths measured by DLS. Data are shown as mean ± SEM, n = 3, * *p* < 0.05, ** *p* < 0.01, *** *p* < 0.001. (**c**) Hydrodynamic sizes intensity distribution of CaA microgels with different flow rate ratio measured by DLS. (**d**) Hydrodynamic particle sizes of CaA microgels with different flow rate ratio measured by DLS. Data are shown as mean ± SEM, n = 3, * *p* < 0.05, ** *p* < 0.01, *** *p* < 0.001. (**e**) Hydrodynamic particle sizes of CaA microgels different channel lengths and flow rate ratios. (**f**) TEM image of CaA microgels. (**g**) The hydrodynamic particle sizes of CaA microgels measured over 77 h using DLS.

For continuous CaA microgels production, the channel length and flow rate ratio play an important role on the particle size. CaA microgels were synthesized by different channel lengths and flow rate ratios (Figure 4). Combining the channel length and flow rate ratio, particle size can be well-controlled which have been confirmed in our experiments (Figure 4a–e).

The effect of channel length on the particle size was investigated under a given flow rate ratio of 0.5. The particle size of CaA microgels measured by dynamic light scattering (DLS) (Figure 4a). The particle size of CaA microgels were gradually increased from 300 nm to 436.5 nm (Figure 4a,b) as the channel lengthened from 30 mm to 100 mm. CaA microgels synthesized by a channel length of 30 mm had the smallest particle size of 300 nm. It can be attributed to the shorter channel length of 30 mm, which underwent less resident time (0.6 s) and mixing efficiency of alginate (31.8%). In the following experiments, a microfluidic chip with a channel length of 30 mm was used for synthesizing CaA microgels.

To investigate the effect of flow rate ratio on particle size, flow rate ratio was studied. When the flow rate ratio increased from 0.5 to 4, the particle sizes of CaA microgels were increased from 327 to 514 nm (Figure 4c,d). Flow rate ratio of 0.5 has the smallest particle size of 327 nm. For a larger flow rate ratio, a narrower central diffusion region can generate bigger mixing efficiency and shorter mixing time. In the following experiments, a flow rate ratio of 0.5 was used for synthesizing CaA microgels.

Transmission electron microscopes (TEM) were used to further study the particle size for comparison. TEM image of CaA microgels synthesized at the channel length of 30 mm and flow rate ratio of 0.5 has shown a uniform particles size of around 448 nm in length and 235 nm in diameter (Figure 4f). In addition, investigation on store time at 4 °C indicates that the particle size of the as-synthesized CaA microgels can keep constant for 77 h (Figure 4g and Figure S8), confirming their good stability.

### 3.5. CaA Microgels for Dox

CaA@Dox microgels were synthesized from 3D-printed concentration-controlled microfluidic chips via a central stream strategy. When the total flow rate was 600 μL/min, Dox aqueous solutions were injected into central inlets at the flow rate of 400 μL/min, and two side streams of 100 μL/min. CaA@Dox microgels were synthesized under different initial Dox concentrations of 25, 50, 100, 200 and 400 μg/mL, which were known as CaA@Dox microgels 1, 2, 3, 4 and 5, respectively.

**Figure 5:**
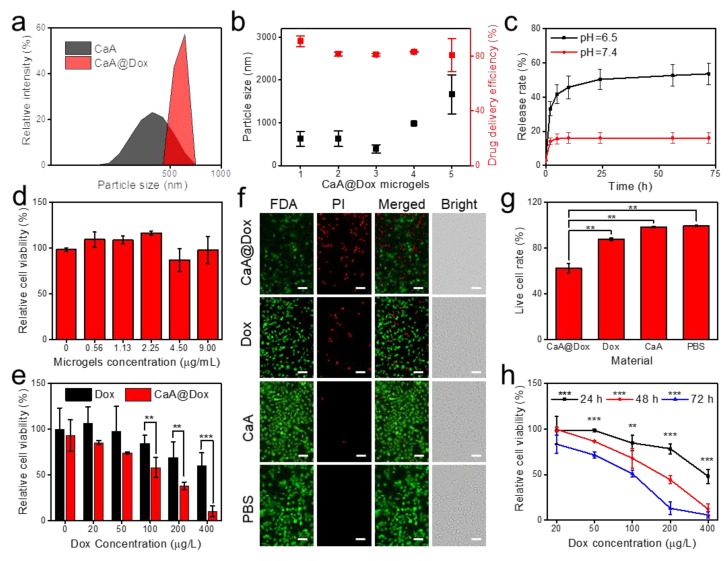
3D-printed concentration-controlled microfluidic chip for Dox delivered CaA microgels (CaA@Dox). (**a**) Particle sizes distribution of CaA microgels and CaA@Dox microgels 3 by DLS. (**b**) Left was particle sizes of CaA@Dox microgels 1–5. Right was drug delivery rate of CaA microgels 1–5. (**c**) Evaluation of the release rate of Dox in CaA microgels 3 under different pH conditions. (**d**) Toxicity assay of CaA microgels 3 under different concentration by CCK-8 kit. The drug administration time was 48h. (**e**) Cytotoxicity of CaA@Dox microgels 1–5 and free Dox with different Dox concentrations against MCF-7 cells for 48 h. Data are shown as mean ± SEM, n = 3, * *p* < 0.05, ** *p* < 0.01, *** *p* < 0.001. (**f**) Fluorescence paragraph for live and dead cell assay of CaA@Dox microgels 3, Dox, CaA microgels and PBS under Dox concentration of 20 μg/L for 24 h. The live cells were dyed by FDA (E_x_/E_m_ = 494/520nm), the dead cells were dyed by PI (488/630 nm). Scale bar was 50 μm. (**g**) Live cell rate calculated by fluorescence paragraph. Data are shown as mean± SEM, n = 3, * *p* < 0.05, ** *p* < 0.01, *** *p* < 0.001. (**h**) Cell survival test of CaA@Dox microgels 3 for 24 h, 48 h, and 72 h on MCF-7. Data are shown as mean± SEM, n = 3, * *p* < 0.05, ** *p* < 0.01, *** *p* < 0.001.

Comparing CaA microgels with CaA@Dox microgels by DLS, the as-synthesized CaA@Dox microgels 1 showed more uniform size distribution and larger particle size (Figure 5a). Figure 5b have shown the particle size of CaA@Dox microgels 1–5. CaA@Dox microgels 3 had the smallest particle size of 388 nm, and CaA@Dox microgels 5 had the largest particle size of 1,665 nm. These experiments demonstrate that the introduction of the drug of Dox can increase the particle size of microgels.

Zeta potential of CaA@Dox microgels 1–5 were used to study the stability of the samples, which were shown in Table S3. All microgels had the negative value of zeta potential that is smaller than −37.7 mV. The more the initial Dox concentration, the less value. It is interesting to note that CaA@Dox microgels 1 of zeta potential of −60.8 mV would come to a condition of excellent stability. Although CaA@Dox microgels 5 had the zeta potential of −37.7 mV, it was still in a good stability.

The Dox delivery efficiency of CaA@Dox microgels 1–5 were investigated (Figure 5b). CaA@Dox microgels 1 had the highest Dox delivery efficiency of 90.7%, and all microgels had the good Dox delivery efficiency over 80%. Compared CaA microgels with CaA@Dox microgels 3 by UV-vis absorption spectra, the as-synthesized CaA@Dox microgels had the significant Dox absorption properties (Figure S9). The aqueous solution color of CaA@Dox microgels exhibited more darker with the increasing initial Dox concentration. It further confirmed that Dox was loaded successfully into the CaA microgels. In consideration of the acidic microenvironment in the solid tumor, the drug release behavior of CaA@Dox microgels was determined at different pH (6.5 or 7.4) [35]. UV-vis absorption spectra of Dox was used to calculate delivery and release efficiency in this study (Figure S10). The results have shown that CaA@Dox microgels 3 at lower pH had the accelerated drug release efficiency (Figure 5c).

The biocompatibility of CaA microgels 3 was studied (Figure 5d). The examination of potential cytotoxicity of pure CaA microgels was cell counting kit (CCK-8) assay, which is widely used for measuring cell viability (Figure 5d). Human breast epithelial cells, MCF-10A, were incubated with CaA microgels at different concentrations for 48 h. The results in Figure 5d revealed that CaA microgels had no obvious toxicity to MCF-10A cells even at high concentration.

As shown in Figure S11a, obvious toxicity was not seen when incubated time increased from 24 to 72 h, and the relative cell viability in all conditions were higher than 75%. The similar biocompatibility results of CaA microgels for MCF-7 cells were provided in Figure S11b. These results demonstrated that CaA microgels synthesized in the concentration-controlled microfluidic chip have good biocompatibility.

More studies were carried out on the chemotherapy effect of CaA@Dox microgels 3 or free Dox against MCF-7 cells by a cell counting kit (CCK-8) assay. MCF-7 cells that were treated with CaA@Dox microgels (final Dox concentration of 400 μg/L) only sustained a cell viability of 10.5% after 48 h. In contrast, MCF-7 cells treated with free Dox of the same concentration sustained a cell viability of 60.2% after 48 h (Figure 5e). This difference can be attributed to the excellent drug delivery efficiency of CaA microgels.

For a better understanding of the chemotherapy effect, the live and dead cells imaging kit was carried out. The MCF-7 cells were incubated with PI and FDA after incubating with samples for 24 h, and then imaged by a fluorescence microscope (Figure 5f). For CaA@Dox microgels, the significant red fluorescence signal of PI obtained in the fluorescence images, which indicated that CaA@Dox microgels have the obvious chemotherapy effect on the MCF-7 cells. For Dox, it had a less red fluorescence signal of PI and a more green fluorescence signal of FDA than CaA@Dox microgels. CaA microgels and PBS control experiments have only the green fluorescence signal of FDA. The live cell rate further confirmed the above results (Figure 5g). All these evidences strongly point to the fact that CaA@Dox microgels can significantly kill the MCF-7 breast tumor cell in vitro.

The chemotherapy effect of CaA@Dox microgels on MCF-7 also have time-dependent and concentration-dependent properties (Figure 5h). As time expanded from 24 to 72 h, the cell viability decreased from 48.3% to 5.8% at a final Dox concentration of 400 μg/L. As concentration of Dox increased from 25 to 400 μg/L after 48 h, the cell viability changed from 99.7% to 12.3%. CaA@Dox microgels had the similar concentration-dependent properties for 12 h and 72 h. These results suggest that the current studies have highlighted the good potential of the CaA@Dox microgels in cancer chemotherapy.

## 4. Conclusions

In summary, a novel concentration-controlled microfluidic chip is successfully designed and 3D-printed for the synthesis of CaA microgels based on the diffusion mixing pattern. The 3D-printed concentration-controlled microfluidic chip exhibits strong channel length- and flow-rate-ratio-dependent particle sizes of CaA microgels in the central stream, which is well aligned with the diffusion mixing pattern from the side stream to the central stream. The excellent drug delivery efficiency also makes the central stream as an idea for a controllable synthesis strategy for drug delivery. Owing to the remarkable biocompatibility, drug delivery and pH-dependent release efficiency of CaA@Dox microgels, satisfying cancer treatment, can be achieved. Most importantly, the proposed diffusion mixing pattern in the 3D-printed concentration-controlled microfluidic chip would provide a simple but controllable synthesis strategy to synthesize CaA microgels, which can helpfully load hydrophilic chemical agents with tunable size. This proof-of-concept about 3D-printed concentration-controlled microfluidic chip with diffusion mixing pattern may be developed as an important synthesis technique to fabricate other kinds of functional nano- and microparticles for drug delivery.

## Supplementary Materials

The following are available online at https://www.mdpi.com/2079-4991/9/10/1451/s1. Table S1: The *R_f_*, *F_c_*, *F_s_*, *t_mix_*, and ME by diffusion in microfluidic channels, Table S2: The channel length, t_res_, and ME by diffusion in microfluidic channels, Table S3: Zeta potential of CaA@Dox at different Dox concentration, Figure S1: Solidwoks draw of the microfluidic chip for generating CaA or CaA@Dox microgel. The microfluidic chip with different length of (a) 30 mm, (b) 40 mm, (c) 50 mm, (d) 100 mm. Scale bar: 10 mm, Figure S2: Fabrication and assembly process of 3D-printed concentration-controlled microfluidic. (a) Model map of chip. (a) 3D-printed chip of different channel length, Figure S3: Channels simulated by COMSOL with channel length of 30 mm (a), 40 mm (b), 60 mm (c), 100 mm (d) and flow rate ratio of 0.5 (e), 1 (f), 2 (g), 4 (h), Figure S4: Simulation experiments to explore effect of chip depth and width. (a) Effect of width and simulation on alginate. (b) Data analysis of simulation on alginate at different depth (0.6, 0.7, 0.8, 1.0 mm). (c) Effect of depth and simulation on alginate. (d) Data analysis of simulation on alginate at different width (0.2, 0.3 mm), Figure S5: Simulation experiments to explore influences by total flow rate (F), Figure S6: Fluorescence intensity analyzed at different *R_f_*, Figure S7: Fluorescence intensity analyzed at different *R_f_*, Figure S8: Size distribution of CaA microgel at different store time at 4 °C, Figure S9: UV spectra (a) and Paragraph (b) of CaA@Dox microgel, Figure S10: (a) Ultraviolet absorption spectrum of pure Dox at different concentration. (b) Standard curve of Dox at 480nm, Figure S11: Cell experiments. (a) Toxicity test of CaA microgel at different concentration for 72h on MCF-10A cells. (b) Toxicity test of CaA microgel at different concentration for 72h on MCF-7 cells.

## References

[1] Chen S., Han Y.H., Wang Y.Q., Yang X., Sun C.X., Mao L.K., Gao Y.X. (2019). Zein-hyaluronic acid binary complex as a delivery vehicle of quercetagetin: Fabrication, structural characterization, physicochemical stability and in vitro release property. Food Chem..

[2] Tsirigotis-Maniecka M., Szyk-Warszynska L., Michna A., Warszynski P., Wilk K.A. (2018). Colloidal characteristics and functionality of rationally designed esculin-loaded hydrogel microcapsules. J. Colloid Interface Sci..

[3] Li X., Li M., Zong L., Wu X., You J., Du P., Li C. (2018). Liquid Metal Droplets Wrapped with Polysaccharide Microgel as Biocompatible Aqueous Ink for Flexible Conductive Devices. Adv. Funct. Mater..

[4] Zykwinska A., Marquis M., Sinquin C., Cuenot S., Colliec-Jouault S. (2016). Assembly of HE800 exopolysaccharide produced by a deep-sea hydrothermal bacterium into microgels for protein delivery applications. Carbohydr. Polym..

[5] Chen D.D., Wu M.D., Chen J., Zhang C.Q., Pan T.Z., Zhang B., Tian H.Y., Chen X.S., Sun J.Q. (2014). Robust, Flexible, and Bioadhesive Free-Standing Films for the Co-Delivery of Antibiotics and Growth Factors. Langmuir.

[6] Eral H.B., Lopez-Mejias V., O’Mahony M., Trout B.L., Myerson A.S., Doyle P.S. (2014). Biocompatible Alginate Microgel Particles as Heteronucleants and Encapsulating Vehicles for Hydrophilic and Hydrophobic Drugs. Cryst. Growth Des..

[7] Wang D., Wang J.Y., Xie W.S., Zhao W., Zhang Y., Sun X.D., Zhao L.Y. (2018). Drug-Loaded Magnetic Microhydrogel as Microwave Susceptible Agents for Cancer Multimodality Treatment and MR Imaging. J. Biomed. Nanotechnol..

[8] Torres A.L., Bidarra S.J., Pinto M.T., Aguiar P.C., Silva E.A., Barrias C.C. (2018). Guiding morphogenesis in cell-instructive microgels for therapeutic angiogenesis. Biomaterials.

[9] Mao A.S., Shin J.W., Utech S., Wang H., Uzun O., Li W., Cooper M., Hu Y., Zhang L., Weitz D.A. (2017). Deterministic encapsulation of single cells in thin tunable microgels for niche modelling and therapeutic delivery. Nat. Mater..

[10] Poncelet D., Babak V.G., Neufeld R.J., Goosen M.F.A., Burgarski B. (1999). Theory of electrostatic dispersion of polymer solutions in the production of microgel beads containing biocatalyst. Adv. Colloid Interface Sci..

[11] Yeung T.W., Ucok E.F., Tiani K.A., McClements D.J., Sela D.A. (2016). Microencapsulation in Alginate and Chitosan Microgels to Enhance Viability of Bifidobacterium longum for Oral Delivery. Front. Microbiol..

[12] Lai W.F., Susha A.S., Rogach A.L. (2016). Multicompartment Microgel Beads for Co-Delivery of Multiple Drugs at Individual Release Rates. ACS Appl. Mater. Interfaces.

[13] Seiffert S. (2013). Small but smart: Sensitive microgel capsules. Angew. Chem. Int. Ed..

[14] Chen K., Li J., Feng Y., He F., Zhou Q., Xiao D., Tang Y. (2017). Structural and rheological characterizations of nanoparticles of environment-sensitive hydrophobic alginate in aqueous solution. Mater. Sci. Eng. C.

[15] Lee K.Y., Mooney D.J. (2012). Alginate: Properties and biomedical applications. Prog. Polym. Sci..

[16] Augst A.D., Kong H.J., Mooney D.J. (2006). Alginate hydrogels as biomaterials. Macromol. Biosci..

[17] Catherine K., Kuo P.X.M. (2001). Ionically crosslinked alginate hydrogels as scaffolds for tissue engineering: Part 1. Structure, gelation rate and mechanical properties. Biomaterials.

[18] Xu X., Zhao L., Xue Q., Fan J., Hu Q., Tang C., Shi H., Hu B., Tian J. (2019). Dynamic Liquid Surface Enhanced Raman Scattering Platform Based on Soft Tubular Microfluidics for Label-Free Cell Detection. Anal. Chem..

[19] Hou L., Ren Y., Jia Y., Deng X., Liu W., Feng X., Jiang H. (2017). Continuously electrotriggered core coalescence of double-emulsion drops for microreactions. ACS Appl. Mater. Interfaces.

[20] Sackmann E.K., Fulton A.L., Beebe D.J. (2014). The present and future role of microfluidics in biomedical research. Nature.

[21] Mao S., Zhang Q., Li H., Zhang W., Huang Q., Khan M., Lin J.M. (2018). Adhesion analysis of single circulating tumor cells on a base layer of endothelial cells using open microfluidics. Chem. Sci..

[22] Wang J., Song Y. (2017). Microfluidic Synthesis of Nanohybrids. Small.

[23] Toth M.J., Kim T., Kim Y. (2017). Robust manufacturing of lipid-polymer nanoparticles through feedback control of parallelized swirling microvortices. Lab Chip.

[24] Feng Q., Liu J., Li X., Chen Q., Sun J., Shi X., Ding B., Yu H., Li Y., Jiang X. (2017). One-Step Microfluidic Synthesis of Nanocomplex with Tunable Rigidity and Acid-Switchable Surface Charge for Overcoming Drug Resistance. Small.

[25] Zhang L., Feng Q., Wang J., Sun J., Shi X., Jiang X. (2015). Microfluidic synthesis of rigid nanovesicles for hydrophilic reagents delivery. Angew. Chem. Int. Ed..

[26] Sun J., Xianyu Y., Li M., Liu W., Zhang L., Liu D., Liu C., Hu G., Jiang X. (2013). A microfluidic origami chip for synthesis of functionalized polymeric nanoparticles. Nanoscale.

[27] Majedi F.S., Hasani-Sadrabadi M.M., Emami S.H., Shokrgozar M.A., VanDersarl J.J., Dashtimoghadam E., Bertsch A., Renaud P. (2013). Microfluidic assisted self-assembly of chitosan based nanoparticles as drug delivery agents. Lab Chip.

[28] Pessoa A., Sipoli C.C., de la Torre L.G. (2017). Effects of diffusion and mixing pattern on microfluidic-assisted synthesis of chitosan/ATP nanoparticles. Lab Chip.

[29] Majedi F.S., Hasani-Sadrabadi M.M., Emami S.H., Taghipoor M., Dashtimoghadam E., Bertsch A., Moaddel H., Renaud P. (2012). Microfluidic synthesis of chitosan-based nanoparticles for fuel cell applications. Chem. Commun..

[30] Kim K., Kang D.H., Kim M.S., Kim K.S., Park K.M., Hong S.C., Chang P.S., Jung H.S. (2015). Generation of alginate nanoparticles through microfluidics-aided polyelectrolyte complexation. Colloids Surfaces A.

[31] Borro B.C., Bohr A., Bucciarelli S., Boetker J.P., Foged C., Rantanen J., Malmsten M. (2019). Microfluidics-based self-assembly of peptide-loaded microgels: Effect of three dimensional (3D) printed micromixer design. J. Colloid Interface Sci..

[32] Bohr A., Boetker J., Wang Y., Jensen H., Rantanen J., Beck-Broichsitter M. (2017). High-Throughput Fabrication of Nanocomplexes Using 3D-Printed Micromixers. J. Pharm. Sci..

[33] Yin P., Hu B., Yi L., Xiao C., Cao X., Zhao L., Shi H. (2018). Engineering of Removing Sacrificial Materials in 3D-Printed Microfluidics. Micromachines.

[34] Jiao Z.Q., Zhao L., Tang C., Shi H.Y., Wang F., Hu B. (2019). Droplet-based PCR in a 3D-printed microfluidic chip for miRNA-21 detection. Anal. Methods.

[35] Mura S., Nicolas J., Couvreur P. (2013). Stimuli-responsive nanocarriers for drug delivery. Nat. Mater..

