# Supplementary material for: 3D-Printed Concentration-Controlled Microfluidic Chip with Diffusion Mixing Pattern for the Synthesis of Alginate Drug Delivery Microgels
Source: Nanomaterials (Basel). 2019 Oct 12;9(10):1451. doi: 10.3390/nano9101451 (PMC6835883; doi:10.3390/nano9101451)
Supplement: Supplementary file 1 [file nanomaterials-09-01451-s001.pdf]

Supporting Information

Article

3D-Printed Concentration-Controlled Microfluidic Chip with Diffusion Mixing Pattern for the Synthesis of Alginate Drug Delivery Microgels

Shixuan Cai [1,#], Hongyan Shi[1,2,#], Guoqian Li [1], Qilu Xue [1], Lei Zhao [1,*], Fu Wang [1,*] *and* Bo Hu [1,*]

1 School of Life Science and Technology, Xidian University, Xi'an 710126, Shaanxi, China
2 Kunpad Communication Pty. Ltd., Kunshan 215300, Jiangsu, China
* Correspondence: zhaolei@xidian.edu.cn (L.Z.); fwang@xidian.edu.cn (F.W.); bohu@xidian.edu.cn (B.H.)
# These authors contributed equally to this work.

Received: 16 August 2019; Accepted: 11 October 2019; Published: date

Table S1. The R_f, Flow Ratio, Re number, and Mixing Time by Diffusion in Microfluidic Channels[a]

Flow rate ratio R_f	Flow rate (central) Fc (μL min^{-1})	Flow rate (side) Fs (μL min^{-1})	Mixing time (t_{mix}) (s)	Mixing efficiency ME
0.5	400	100	49.38	0.53
1	100	150	27.78	0.39
2	200	200	12.35	0.29
4	120	240	4.44	0.23

[a]Channel length maintain at 40 mm.

Table S2. The Length, R_f, Re Number, and Mixing time by Diffusion in Microfluidic Channels[a]

Channel length (mm)	Resident time (t_{res}) (s)	Mixing efficiency ME
30	0.6	0.32
40	0.8	0.36
50	1	0.40
100	2	0.52

[a]Flow rate ratio maintain at 2.

Table S3. Zeta Potential of CaA @ Dox at Different Dox Concentration

Concentration (ug/mL)	Zeta potential (mV)

25	-60.83
50	-46.95
100	-23.37
200	-24.64
400	-37.70

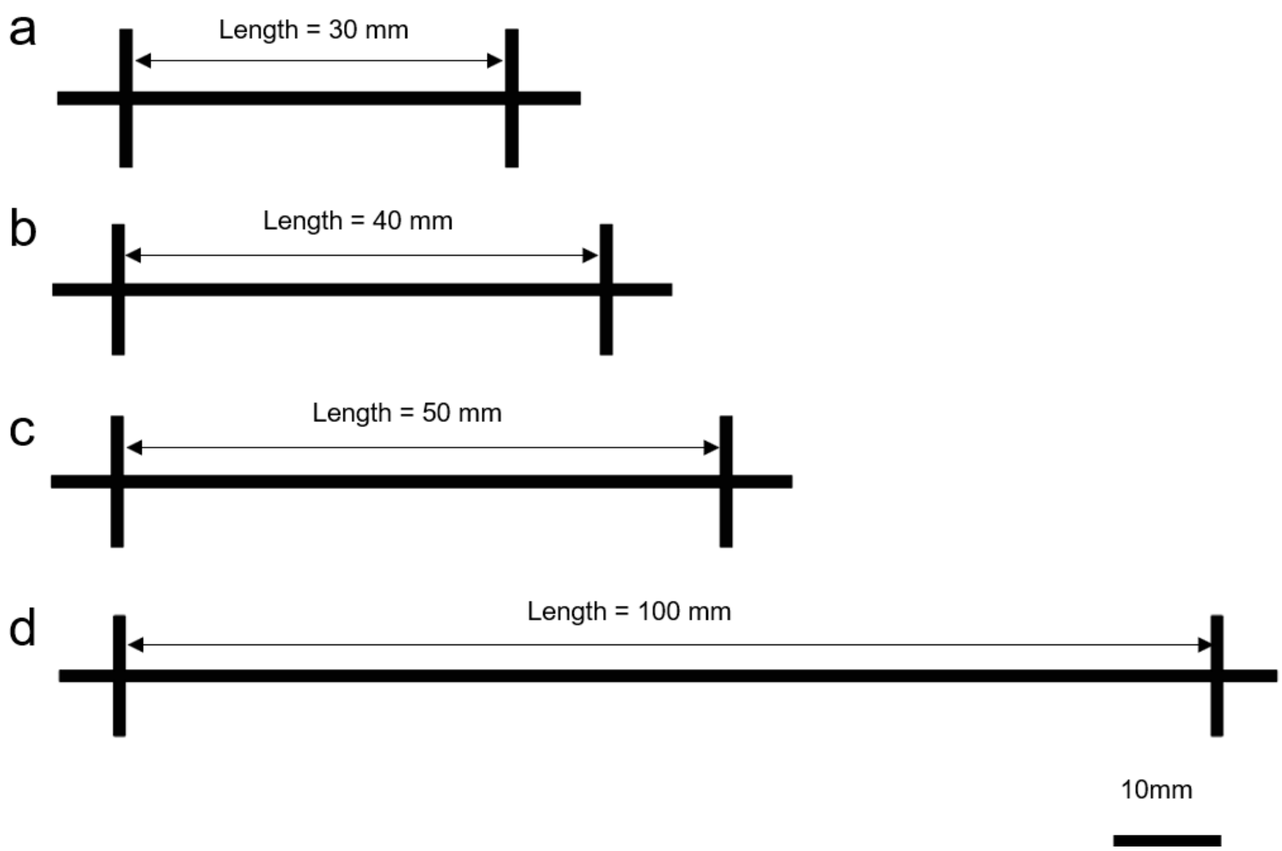

FigureS1. Solidwoks draw of the microfluidic chip for generating CaA or CaA@DOX microgel. The microfluidic chip with different length of (a) 3mm, (b) 4mm, (c) 5mm, (d) 10mm. Scale bar, 10mm.

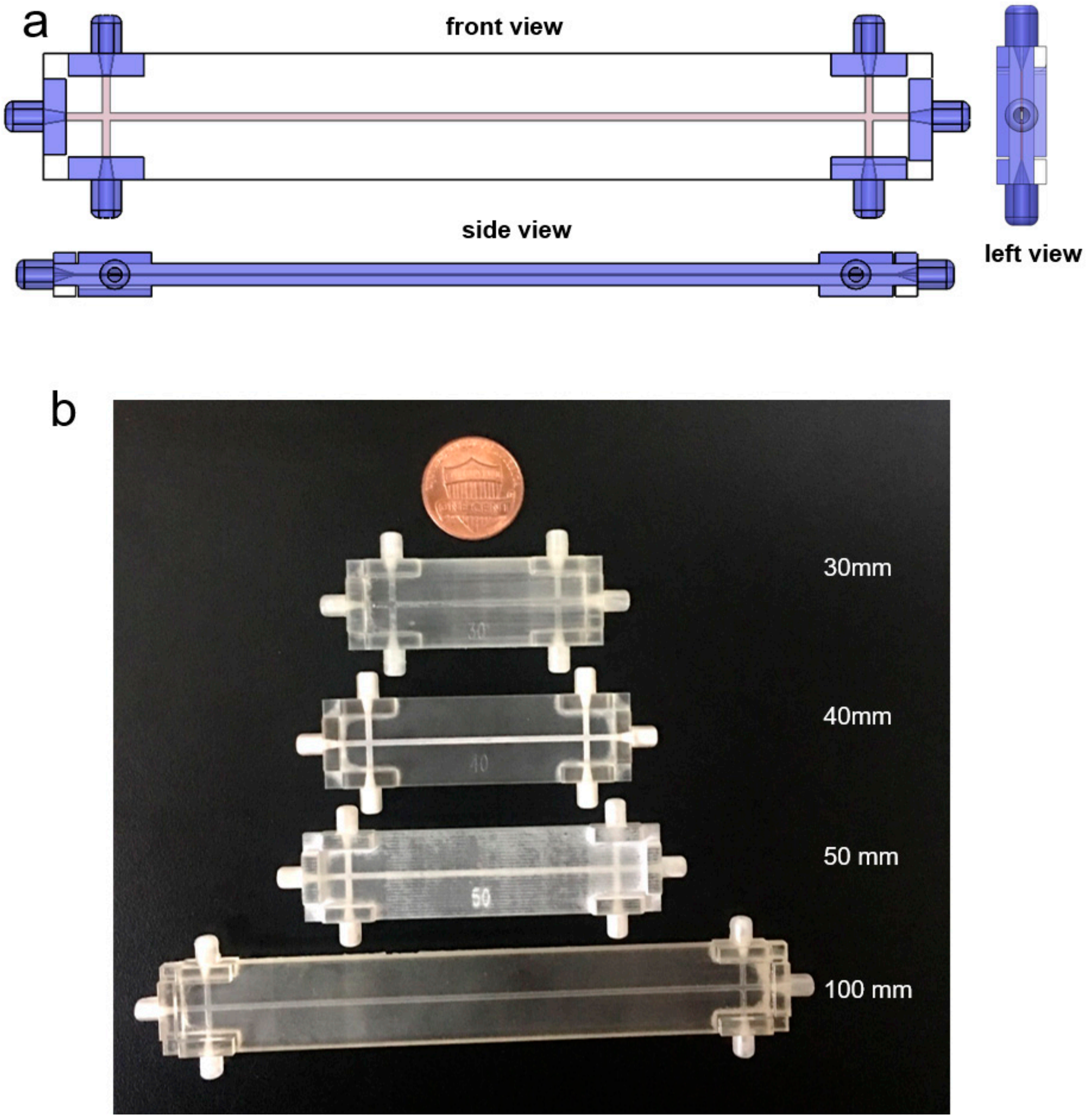

FigureS2. Fabrication and assembly process of 3D-printed concentration-controlled microfluidic. a. Model map of chip. b. 3D-printed chip of different channel length.

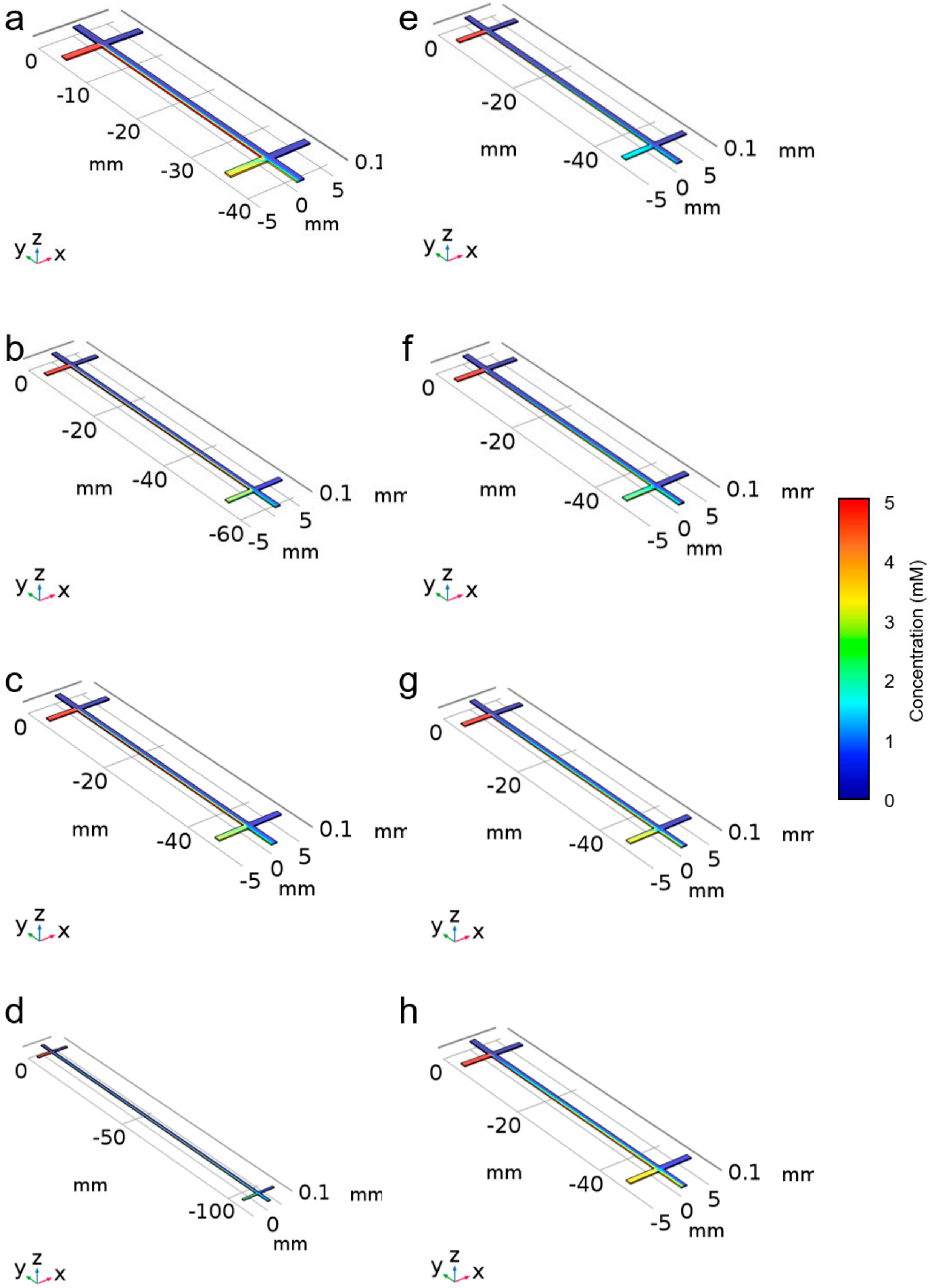

Figure S3. Channels simulated by COMSOL with different channel length (a, b, c and d) and flow rate ratio (e, f, g and h).

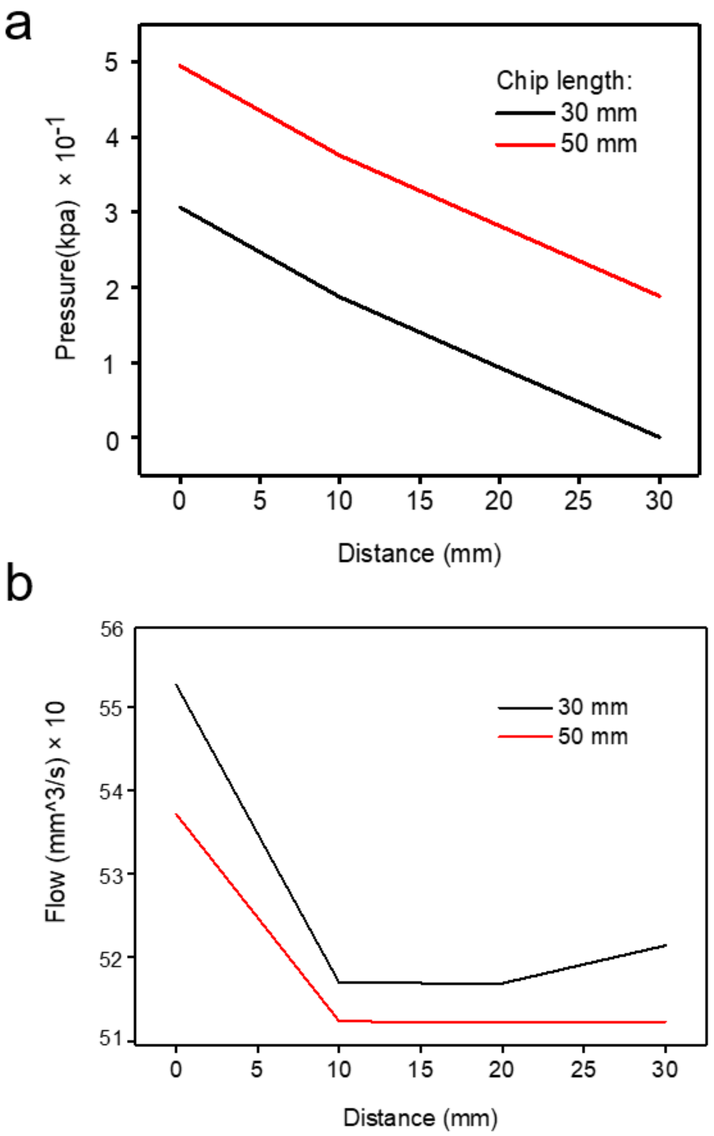

Figure S4. Simulation of pressure (a) and flow rate (b) at different situation.

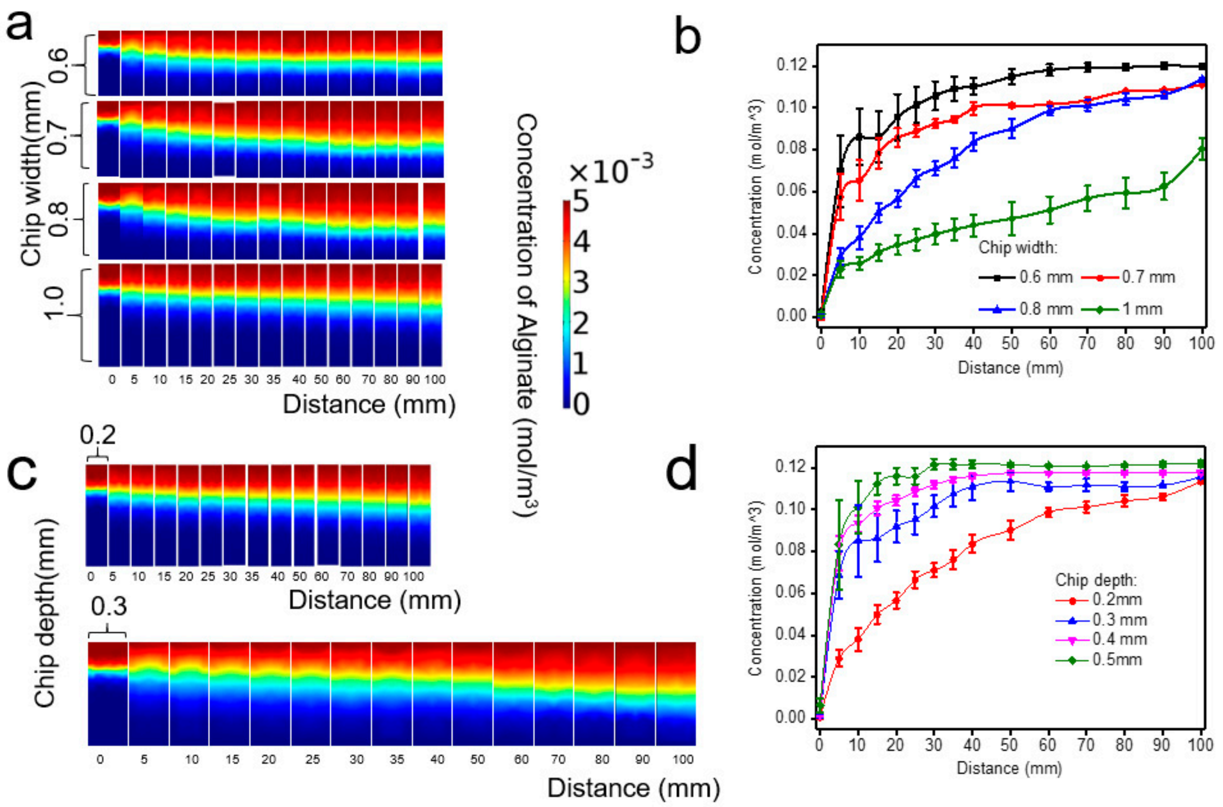

Figure S5. Simulation experiments to explore effect of chip depth and width. a. Effect of width and simulation on alginate. b. Data analysis of simulation on alginate at different depth (0.6、0.7、0.8、1.0 mm). c. Effect of depth and simulation on alginate. d. Data analysis of simulation on alginate at different width (0.2、0.3、0.4、0.5 mm).

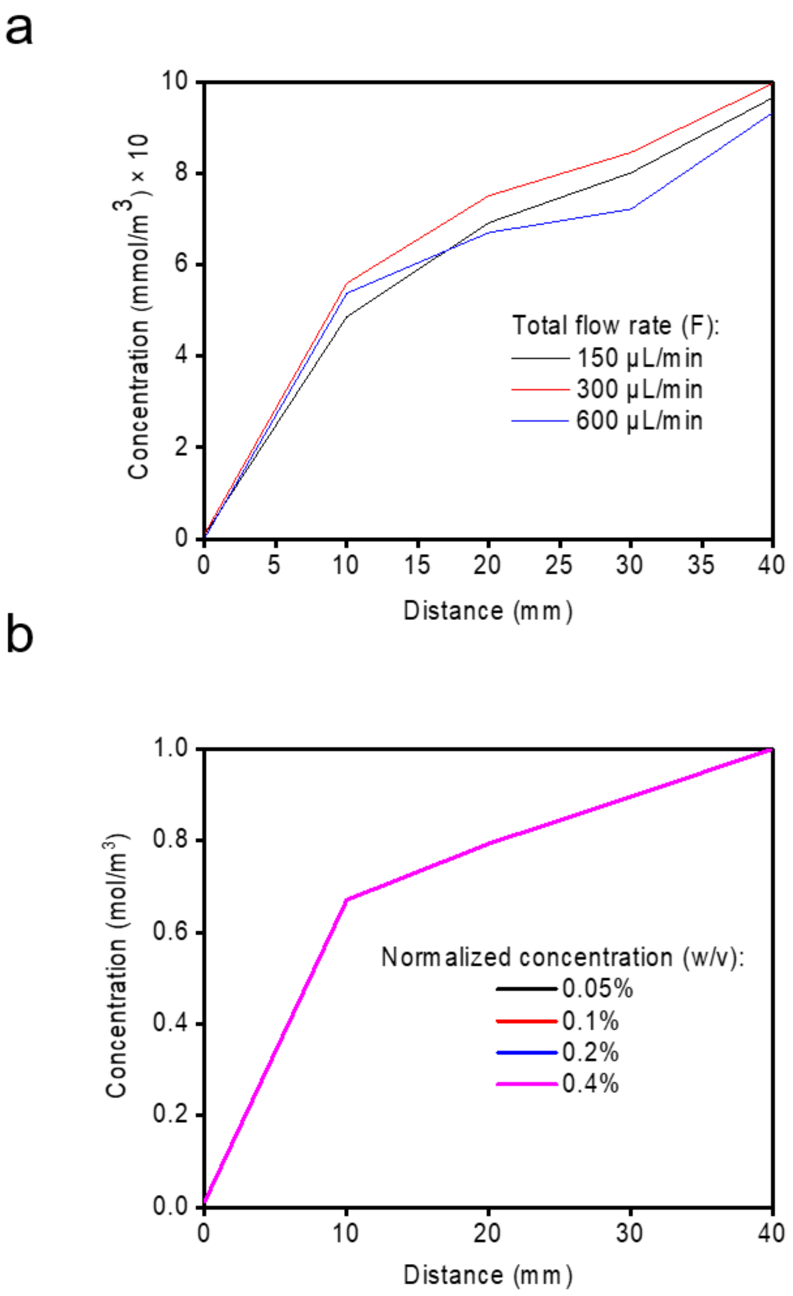

Figure S6. Simulation experiments to explore influences by different factors. a. Affected by total flow rate (F). b. Affected by concentration of alginate (Rc).

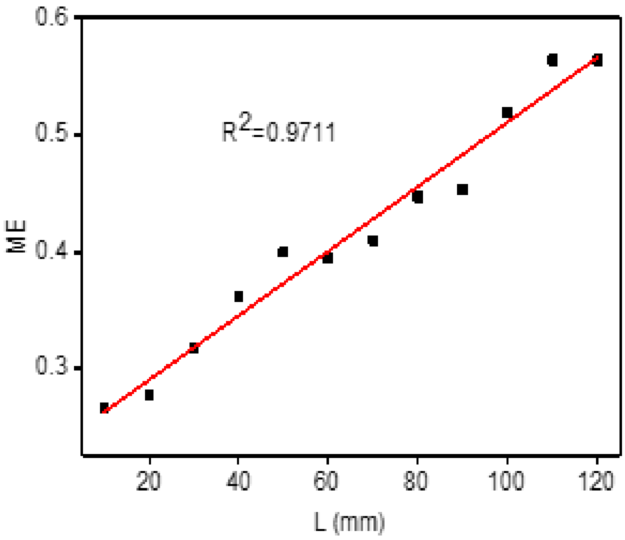

Figure S7. Relative between channel length and mixing efficiency.

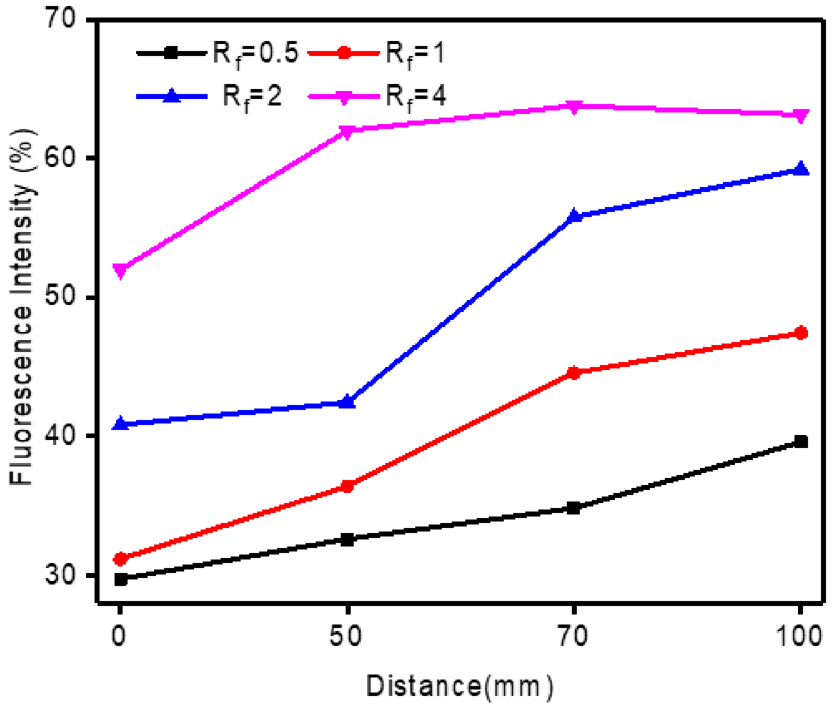

Figure S8. Fluorescence intensity analyzed at different R_f.

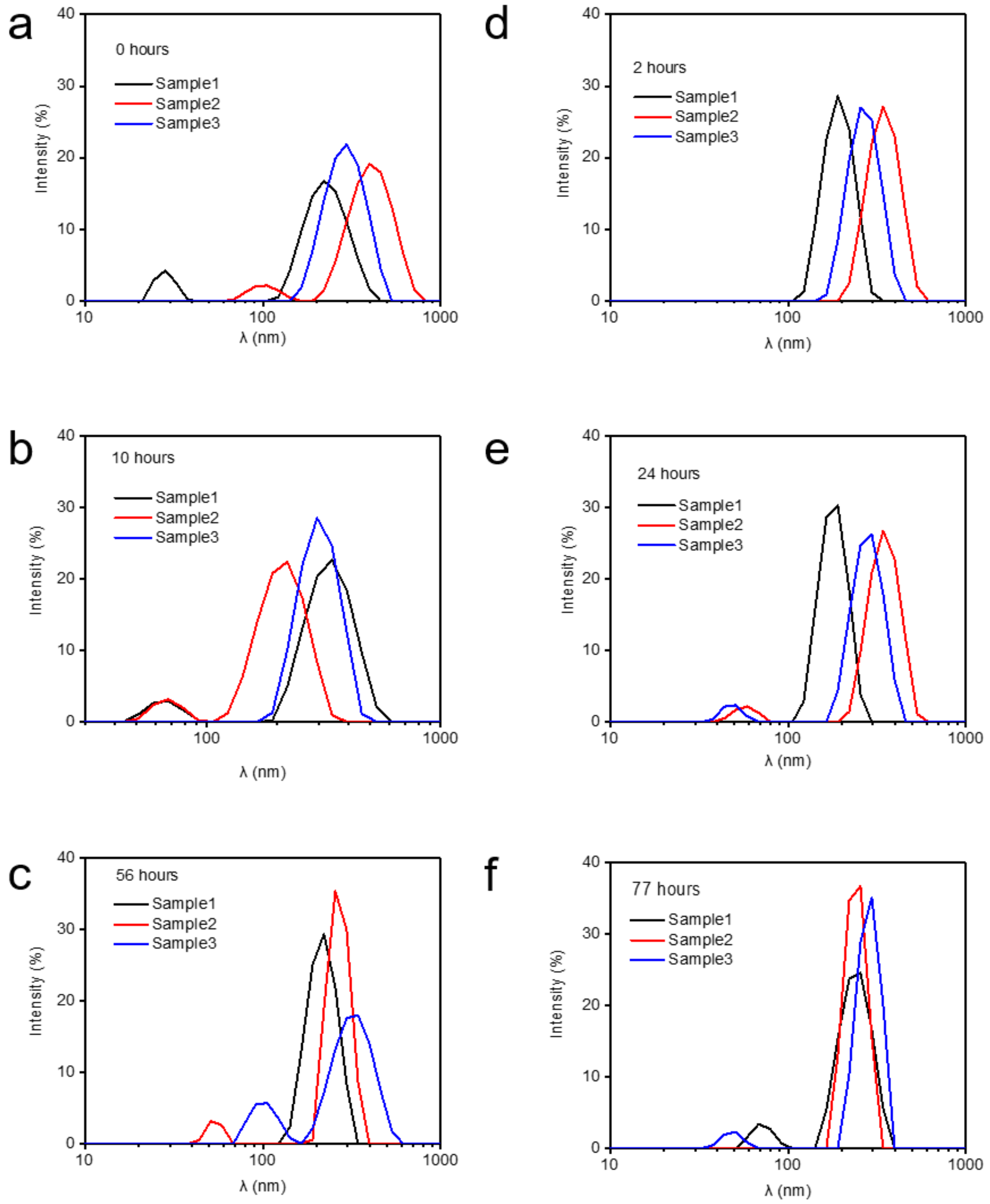

Figure S9. Size distribution of CaA microgel at different store time at 4 °C.

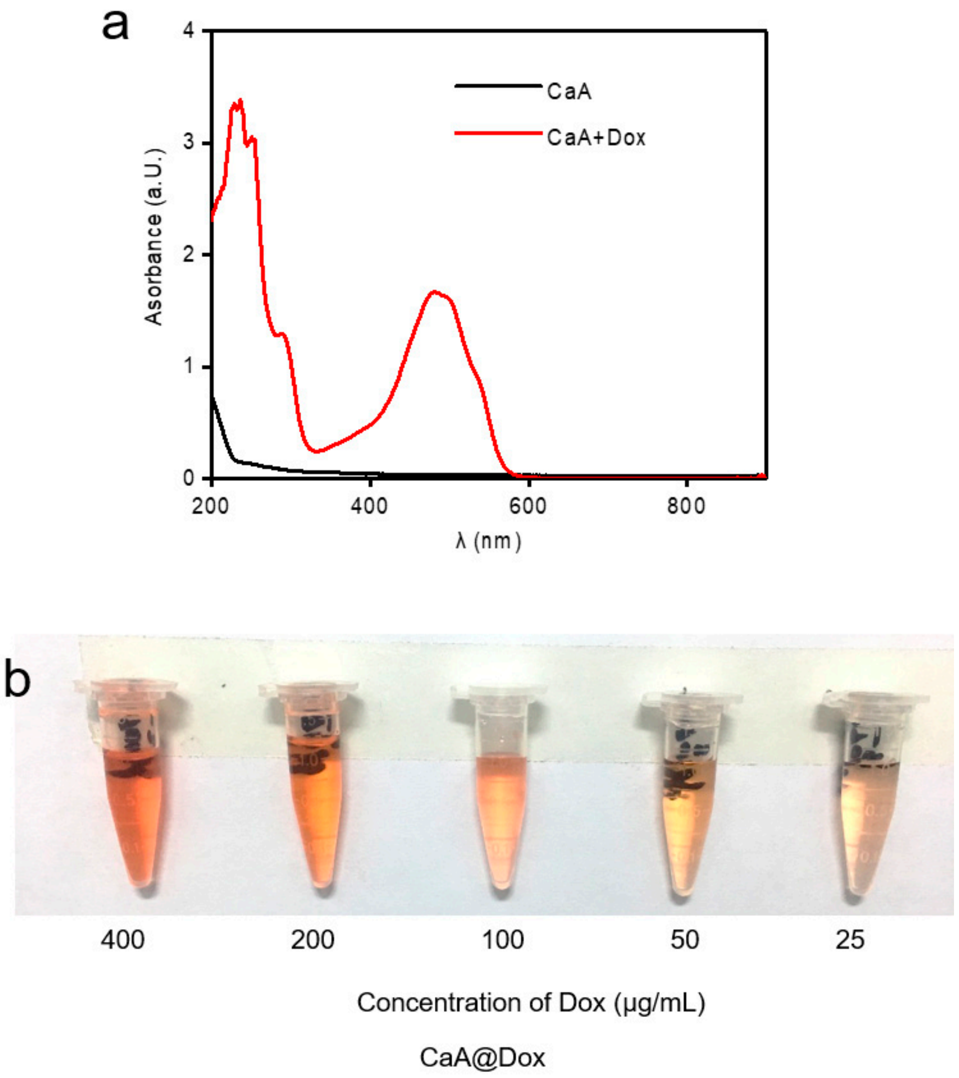

Figure S10. UV spectra (a) and Paragragh (b) of CaA@Dox microgel.

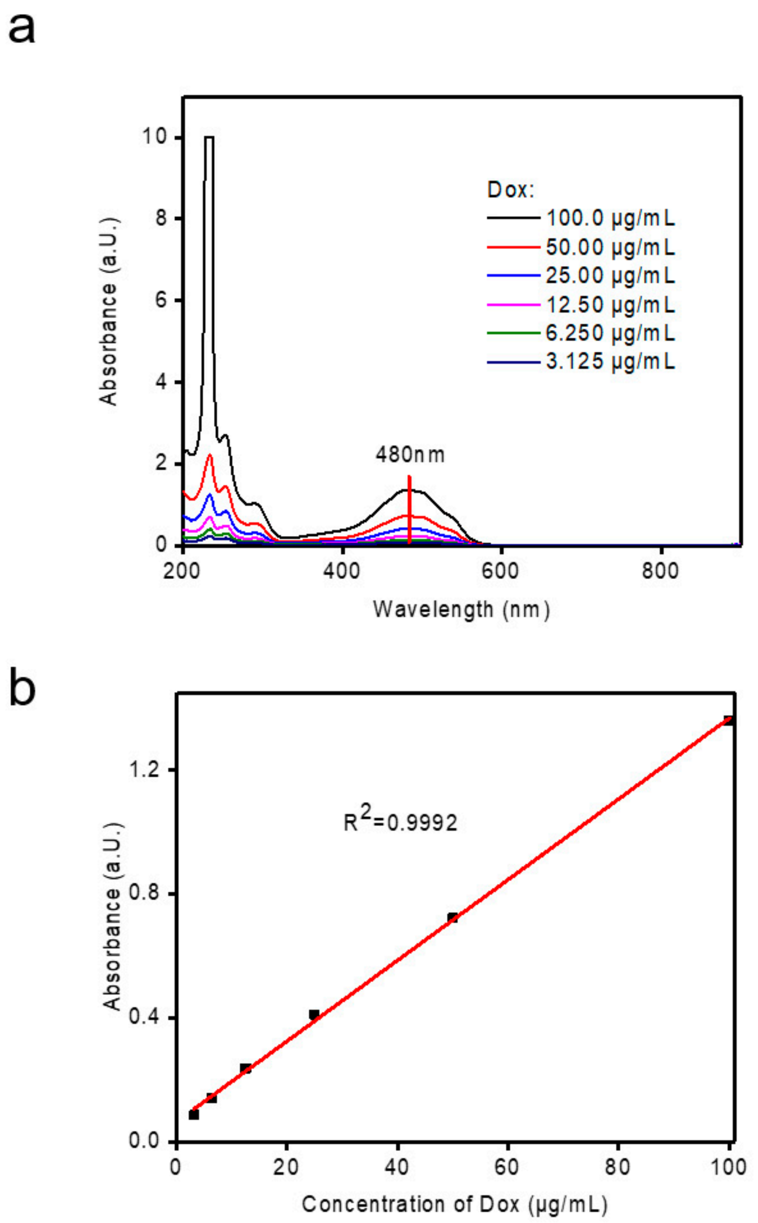

Figure S11. a. Ultraviolet absorption spectrum of pure Dox at different concentration. b. Standard curve of Dox at 480nm.

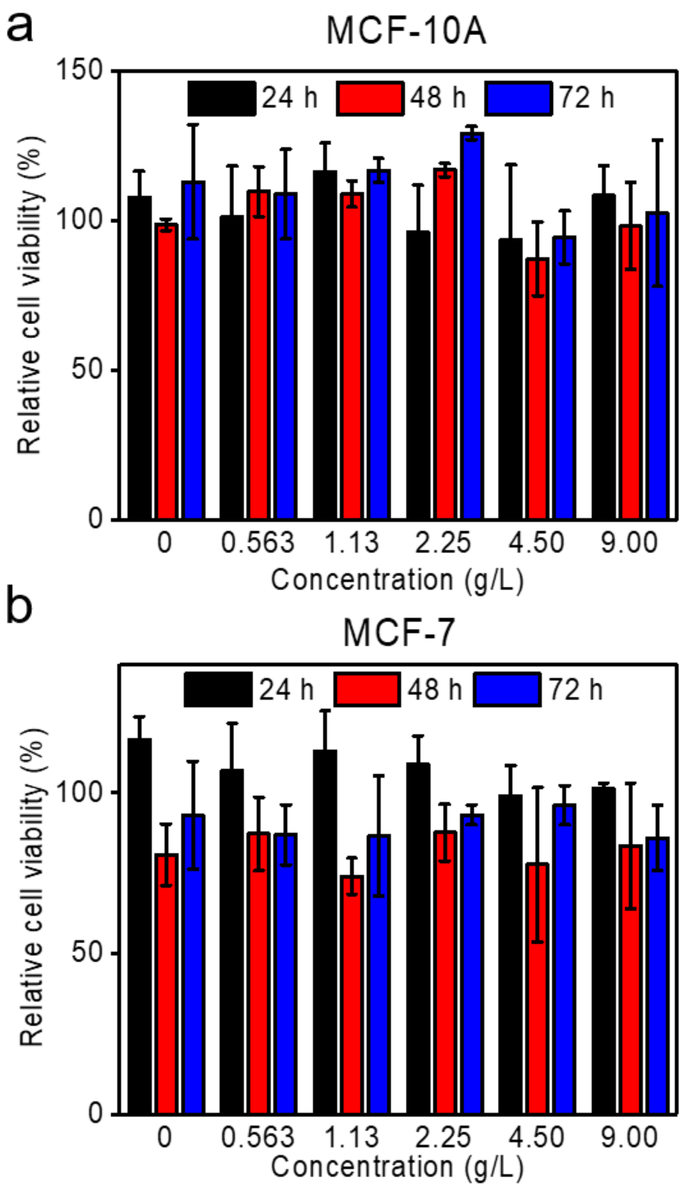

Figure S12. Cell experiments. a. Toxicity test of CaA microgel at different concentration for 72h on MCF-10A cells. b. Toxicity test of CaA microgel at different concentration for 72h on MCF-7 cells.
